# A very low carbohydrate diet improved metabolic profile in congenital generalized lipodystrophy type 4

**DOI:** 10.1530/EDM-24-0063

**Published:** 2025-01-27

**Authors:** Sayantan Chakraborty, Subhankar Roy, Debaditya Das, Sayantani Chatterjee, Pranab Kumar Sahana, Satinath Mukhopadhyay

**Affiliations:** Department of Endocrinology, IPGME&R, Kolkata, India

**Keywords:** Congenital Generalized Lipodystrophy, Hypertriglyceridemia, Low Carbohydrate diet, Insulin resistance

## Abstract

**Summary:**

A 17-year-old girl presented with recurrent attacks of acute pancreatitis, associated with severe hyperglycemia and hypertriglyceridemia, despite being on intensive insulin therapy for the last 10 years. She had severe acanthosis nigricans, generalized loss of subcutaneous fat and prominent veins over extremities. The serum levels of glucose and triglyceride did not reduce significantly, even with maximally tolerated doses of metformin (2 g), pioglitazone (45 mg) and fenofibrate (160 mg), not uncommonly seen in poor rural families in West Bengal, India. A detailed dietary recall revealed a very high carbohydrate intake (70% of total calorie) with very low protein and fat intake. A switch to a very low carbohydrate (30% of total calorie) diet led to a remarkable improvement in glucose and lipid profiles (the daily insulin requirement came down by 50% and triglyceride level came down to 600 mg/dL from 950 mg/dL). A whole-exome sequencing study confirmed congenital generalized lipodystrophy type 4. A carbohydrate restriction strategy may improve difficult-to-control glycometabolic profile in lipodystrophic subjects on high-carbohydrate diet.

**Learning points:**

- Lipodystrophy should be suspected in patient presenting with hyperglycemia, hypertriglyceridemia and low BMI.
- A very low carbohydrate diet (30% of total daily calorie intake) may significantly improve glucose and lipid profiles in patients with lipoatrophic diabetes.
- Blood glucose may be the most important initial step to control hypertriglyceridemia and risk of pancreatitis in this group of patients.

## Background

Lipodystrophy is a rare group of disorders characterized by complete or partial loss of subcutaneous fat in the absence of nutritional deprivation or a catabolic state. It is broadly divided into congenital and acquired variants, each having generalized and partial forms based on the pattern and distribution of fat loss. Congenital generalized lipodystrophy is a variant of lipodystrophic syndrome characterized by uncontrolled hyperglycemia, generalized lipoatrophy and hypertriglyceridemia. Congenital generalized lipodystrophy type 4 (CGL4) is a rare form of lipodystrophy associated with a loss-of-function mutation in the *CAVIN1* gene. Here, we report the case of a 17-year-old girl presenting with severe insulin resistance, uncontrolled hyperglycemia and generalized lipodystrophy despite being on maximally tolerated medical treatment. Reducing her carbohydrate intake from 70 to 30% resulted in significant improvement in insulin resistance, hyperglycemia and hypertriglyceridemia. The case is reported because of its relative rarity and challenges in its management.

## Case presentation

A 17-year-old female presented with uncontrolled hyperglycemia. She had a history of diabetes for the last ten years and was on insulin therapy (twice daily premix regimen). She also had a history of recurrent abdominal pain with bulky, fatty stools and was hospitalized thrice with acute pancreatitis in the last three years. She was born out of a non-consanguineous marriage. The birth history was uneventful. There was no history of chronic heart or kidney disease; her menstrual cycles were predictable and regular.

## Investigations

**Figure 1:**
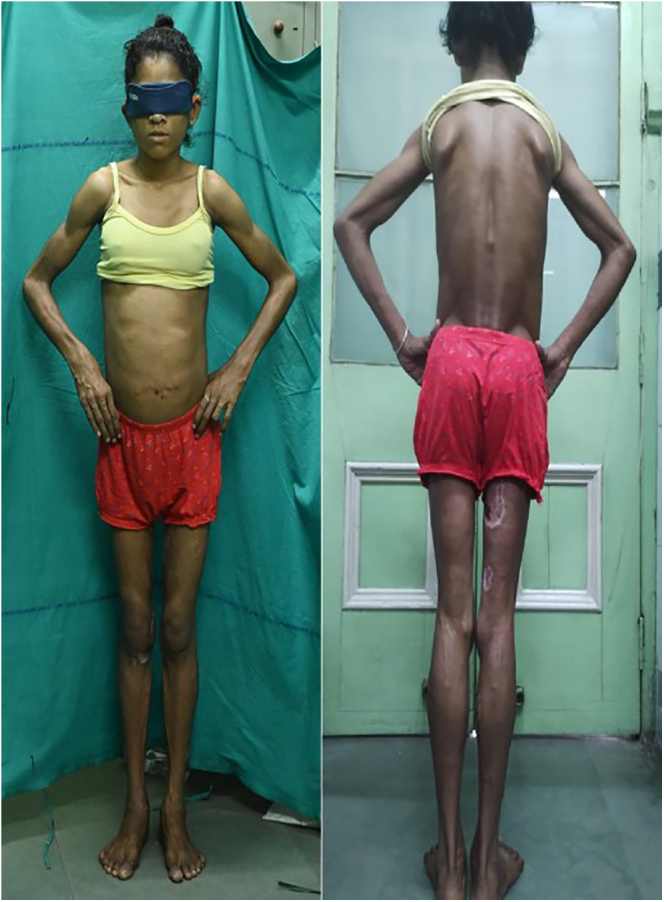
Generalised loss of fat and prominent veins and muscles over extremity.

On examination, she was lean (BMI 16 kg/m^2^), had acanthosis nigricans over the nape of the neck, had generalized loss of subcutaneous fat, had prominent veins over extremity (Fig. 1) and had a scar mark over the umbilicus indicating previous surgery. She was admitted with uncontrolled hyperglycemia (fasting plasma glucose: 350 mg/dL, post-meal plasma glucose: 478 mg/dL, HbA1c: 12.8% (116 mmmol/mol)) and dyslipidemia (fasting serum triglyceride level: 1,100 mg/dL, Table 1). An ultrasonography of the whole abdomen revealed a grade 2 fatty liver. A dual energy X-ray absorptiometry revealed a severely depleted total body fat content (7%) and lean body mass (89%). A whole-exome sequencing revealed a novel homozygous deletion (674 bp) in exon-1 overlapping contiguous region encompassing the *CAVIN1* gene, consistent with CGL4.

**Table 1:** Laboratory parameters.

Parameters	Values
Hemoglobin, g/dL	11.5
Fasting plasma lipid profile	
LDL, mg/dL	95
HDL, mg/dL	25
TG, mg/dL	1,100
On intensifying insulin	950
After very low CHO diet	600
Serum urea, mg/dL	20
Serum creatinine, mg/dL	0.5
Spot urine AL/CR ratio, μg/mg	26
Electrocardiogram	Within normal limit
2D echo	Normal study; LVEF-64%
Serum AST, U/L	33
Serum ALT, U/L	43
Ultrasonography whole abdomen	Grade II fatty liver, bulky pancreas
Serum LH, mIU/mL	3.88
Serum FSH, mIU/mL	6.55
DEXA scan	
Lean body muscle mass, kg (%)	28.552 (89.3)
Total body fat, %	8.3
Serum CPK, μg/L	89
Reference range, μg/L	10–120

AL, albumin; CHO, carbohydrate; CR, creatinine; TG, triglyceride.

## Treatment

Upon hospitalization, her glucose levels continued to remain above the acceptable limits despite high-dose continuous intravenous insulin therapy (04 units/kg/day) along with metformin (2 g/day) and pioglitazone (45 mg/day). Fasting serum triglyceride levels could not be brought under control despite maximally tolerated doses of fenofibrate (160 mg). A relook into the dietary history revealed a very high carbohydrate (70% of total calories) and a very low protein intake (10% of total calories). She was transferred to a very low carbohydrate (30% of total calories) diet (Table 2).

**Table 2:** Dietary changes from high carbohydrate to low carbohydrate and high fat diet.

	Dietary intake at presentation	Modified very low carbohydrate diet
Total calorie intake per day	1,526 kcal	1,750 kcal
Carbohydrate intake	267 g (70%)	131 g (30%)
Protein intake	38 g (10%)	87.5 g (20%)
Fat intake	33 g (20%)	97 g (50%)

## Outcome

A week of supervised medical nutrition therapy led to normalization of blood glucose levels, with a 50% reduction in total daily insulin requirement (from 4 to 2 units/kg/day), associated with a decrease in the serum triglyceride levels (from 950 to 600 mg/dL).

## Discussion

Congenital generalized lipodystrophy is an autosomal recessive that are classified into four types based on gene mutation. The altered genes play essential functions for adipocyte formation, lipid production and proper storage inside the adipocyte. The mutations decrease adipose tissue with consequent deposition of fat in ectopic sites, causing fatty liver, altered carbohydrate metabolism, severe insulin resistance with hyperinsulinemia, acromegaloid features and dyslipidemia. Types 1 and 2 are responsible for over 95% of cases. CGL type 2 has a more severely affected phenotype. Only one case of CGL type 3 and around 30 cases of CGL type 4 have been reported till date in the literature. CGL type 4 is a rare type of lipidystrophy in which the affected gene is the *CAVIN1*, which encodes the protein CAVIN1. CAVIN, a caveolae-associated protein, is an essential component of caveolae, which are invaginations of the cell surface membrane in the fat cells. An absence of caveolin results in significant elevation of serum triglycerides and free fatty acids, and dramatically increased levels of insulin. They also exhibit reduced adiponectin expression and are characteristic of lipodystrophy with insulin resistance (1). Loss of caveolin-1 dampens insulin receptor signaling, leading to unregulated lipolysis and a reduction of lipogenic signals (2). In humans, it has been reported in patients with congenital generalized lipodystrophy and muscular dystrophy (3). Mutations in polymerase I and transcript release factor (*PTRF/CAVIN1*) were reported in five Japanese patients presenting with myopathy and CGL (CGL4). All patients had near total loss of body fat and congenital myopathy manifesting as weakness, and high serum creatine kinase levels like our patient (4). A similar case of severe insulin resistance syndrome with atrial septal defect was reported from our institute (5). India is predominantly a carbohydrate-consuming country. According to the multicenter STARCH studies performed in five different regions of India, the average daily carbohydrate intake is 64.1% (6). This patient was also consuming the same. After dietary modification to low-carbohydrate and high-protein diet, her blood glucose and triglyceride levels gradually came down along with reduction in insulin requirement. One limitation in this case is not documenting any change in the total body fat and liver fat, which will be done in subsequent visits.

## Declaration of interest

The authors declare that there is no conflict of interest that could be perceived as prejudicing the impartiality of the work reported.

## Funding

This work did not receive any specific grant from any funding agency in the public, commercial or not-for-profit sector.

## Patient consent

Written consent has been obtained from each patient or subject after full explanation of the purpose and nature of all procedures used.

## Author contribution statement

All authors made individual contributions to authorship. SC, SS and SR were involved in the management of the patient. DD was involved in diagnosis. PKS and SM were involved in monitoring the response and also supervising the manuscript. All authors reviewed and approved the final draft.
